# A Novel Type I Interferon Primed Dendritic Cell Subpopulation in TREX1 Mutant Chilblain Lupus Patients

**DOI:** 10.3389/fimmu.2022.897500

**Published:** 2022-07-13

**Authors:** Anne Eugster, Denise Müller, Anne Gompf, Susanne Reinhardt, Annett Lindner, Michelle Ashton, Nick Zimmermann, Stefan Beissert, Ezio Bonifacio, Claudia Günther

**Affiliations:** ^1^ Center for Regenerative Therapies Dresden, Faculty of Medicine Technische Universität (TU), Dresden, Germany; ^2^ Center for Molecular and Cellular Bioengineering (CMCB), DRESDEN-Concept Genome Center Technische Universität, Dresden, Germany; ^3^ Department of Dermatology, Faculty of Medicine, University Hospital Carl Gustav Carus, Technische Univeristät Dresden, Dresden, Germany; ^4^ Faculty of Medicine, Paul Langerhans Institute Dresden of Helmholtz Centre Munich at University Clinic Carl Gustav Carus, Technische Universität Dresden, Dresden, Germany

**Keywords:** monogenic familial chilblain lupus, SLE, Type I interferons, dendritic Cells (DC), LMNA, Lamin A/C, Trex1

## Abstract

Heterozygous TREX1 mutations are associated with monogenic familial chilblain lupus and represent a risk factor for developing systemic lupus erythematosus. These interferonopathies originate from chronic type I interferon stimulation due to sensing of inadequately accumulating nucleic acids. We here analysed the composition of dendritic cell (DC) subsets, central stimulators of immune responses, in patients with TREX1 deficiency. We performed single-cell RNA-sequencing of peripheral blood DCs and monocytes from two patients with familial chilblain lupus and heterozygous mutations in TREX1 and from controls. Type I interferon pathway genes were strongly upregulated in patients. Cell frequencies of the myeloid and plasmacytoid DC and of monocyte populations in patients and controls were similar, but we describe a novel DC subpopulation highly enriched in patients: a myeloid DC CD1C^+^ subpopulation characterized by the expression of LMNA, EMP1 and a type I interferon- stimulated gene profile. The presence of this defined subpopulation was confirmed in a second cohort of patients and controls by flow cytometry, also revealing that an increased percentage of patient’s cells in the subcluster express costimulatory molecules. We identified a novel type I interferon responsive myeloid DC subpopulation, that might be important for the perpetuation of TREX1-induced chilblain lupus and other type I interferonopathies.

## Introduction

Chronic uncontrolled immune stimulation by alarming cytokines can break tolerance or hamper silencing of immune responses, leading to autoimmunity. Type I interferons (IFN) induce an antiviral state in immune cells and stimulate dendritic cell differentiation in an immunogenic rather than tolerogenic manner are potential mediators in various autoimmune diseases and especially in systemic lupus erythematosus (SLE) (1) as well as in type I interferonopathies (2). Type I interferonopathies are monogenic diseases characterized by chronic type I interferon activation (3). The first mutations were found in the three prime repair DNA exonuclease 1 (TREX1) and cause monogenic familial chilblain lupus (FCL) (4), Aicardi Goutières syndrome (3), a type I interferonopathy with features of autoimmunity, and represent a risk factor for the development of SLE (5). Defects in TREX1 cause an accumulation of DNA in the cell that can be sensed by the cyclic GMP-AMP synthase (cGAS)-stimulator of IFN genes (STING) pathway leading to chronic type I IFN activation (6, 7). TREX1 deficient mice succumb from autoimmune myocarditis, type I IFN upregulation and cutaneous lesions reminiscent of lupus. This phenotype can be induced by TREX1 deficiency in dendritic cells (DCs), suggesting an important role for DCs as a disease-initiator for this type I interferon driven autoimmune disease (8).

DCs are a heterogeneous population of antigen-presenting cells that orchestrate adaptive immune responses. Various DC subtypes with unique functions reside in all parts of the human body. Blood DC subtypes have classically been defined as CD11c^+^ conventional DCs (cDCs), consisting of either CD141^+^ (cDC1) or CD1c^+^ (cDC2) cells, and CD123^+^ plasmacytoid DCs (pDCs) (9). cDC1s are specialized in fighting intracellular pathogens, while cDC2s are involved in the adaptive immune response towards extracellular pathogens *via* Th1 cell activation by presenting antigens to CD4^+^ T cells (10). Single-cell RNA-sequencing (scRNA-seq) combined with cytometry has further revealed new compartments of human blood DCs, monocytes and progenitors (10–12). The role of these DC populations in disease has not yet been well elucidated and led us to analyse in detail the composition and properties of DCs from patients with familial chilblain lupus and TREX1 deficiency.

## Materials and Methods

### Subjects and PBMC Isolation

Human samples from 3 patients with familial chilblain lupus and a heterozygous mutation in TREX1 (H195Q or D18N) (**Supplemental Table 1** for details on patients) and from 5 age and sex matched Caucasian healthy donors were obtained for the isolation of peripheral blood mononuclear cells (PBMC). PBMC were isolated by density centrifugation. Use of the buffy coats was approved by the ethics committee and informed consent of the donors was obtained (EK 169052010). The study was approved by the ethics committee and informed consent of the donors was obtained (EK 169052010). Patients were involved in the dissemination plans of our research.

### Isolation of Monocytes and Dendritic Cells

For isolation of cells for scRNAseq, freshly isolated PBMC were FC block- treated (2 ul FC blocking reagent human (Miltenyi Biotech) and 48 ul staining buffer (1% BSA in PBS, filtered through a 40 um filter)/1x10^6^ cells) and incubated 10’ on ice. Cells were washed and stained in 50 ul final/1x10^6^ cells of antibody cocktail (CD3-APC (HIT3α, BD), CD19-APC (SJ25C1, BD), CD14-PE-Cy7 (M5E2, BD), CD56-PE-Cy7 (B159, BD), HLA-DR-APC-H7 (B159, BD), CD11c-AF700 (3.9, eBioscience), CD123-BV650 (6H6, Biolegends), CD16-BV605 (3G8, Biolegends)). Life- dead staining with 7AAD (BD) was done shortly before sorting on a FACS (ARIAII, BD) with the 100 uM nozzle using gating strategy shown in **Supplemental Figure 1**. For isolation of cells for qPCR, the same panel was used including also EMP1-FITC (Biozol) (**Supplemental Figure 4A**).

### Intracellular Staining for Analytical Flow Cytometry

For flow cytometric analysis, cells were FC-block treated as described above and then first stained with extracellular antibodies described above in addition to EMP1-FITC (Biozol) and then with viability dye efluor 506 (eBioscience) followed by permeabilization using the eBioscience™ Foxp3/Transcription Factor Staining Buffer Set and staining with LAMIN-FITC (636, Santa Cruz). For staining of activation surface markers, the same panel was used, adding CD40-eFluor 405 (5C3, Thermo Fisher), CD80-PE/Dazzle (2D10, Biolegend) and CD86-BV650 (IT2.2, Biolegend) but omitting CD123-BV650. Measurements were done on FACS (ARIA Fusion, BD) using gating strategy shown in **Supplemental Figure 4A, B**. FMO controls were used in all Flow Cytometry experiments.

### scRNAseq by 10x Genomics

For scRNAseq, 1000 cells from each cell type (CD14^+^ Monocytes, CD11c^+^ DC and pDC) were FACS sorted into 1 ul PBS in the same coated 1.5 ml tube (coated by filling with 1% BSA, by incubating overnight and complete removal of BSA and pre-lying 1 µl PBS). Sorted cells were immediately processed for reverse transcription and library preparation according to the 10x Genomics Protocol using the 10x Genomics Single Cell v2 kit. Libraries were sequenced on a complete Illumina NextSeq 500 high-output flowcell in PE mode (R1: 26 cycles; I1: 8 cycles; R2: 57 cycles. Raw sequencing data was processed with the Cell Ranger software (v2.1.0) provided by 10X Genomics. The human genome (hg38) as well as gene annotations (v87) were downloaded from Ensembl and the annotation was filtered with the ‘mkgtf’ command of Cell Ranger (–attribute=gene_biotype:protein_coding –attribute=gene_biotype:lincRNA –attribute=gene_biotype:antisense). Genome sequence and filtered gene annotation were used as input to build the appropriate Cellranger reference. Cells were removed if expressing fewer than 400 unique genes, more than 4,500 unique genes, or greater than 15% mitochondrial reads. Genes not detected in any cell were removed from subsequent analysis. Downstream analysis was conducted with Seurat V2.4 package in R (3.5.0) (13).

### Multiplex qPCR by Biomark

1000 CD11c^+^, 1000 CD14^+^ cells or 500 CD11c^+^EMP1^+^ cells from 2 patients and 2 controls were FACS sorted in triplicates into PCR tubes containing 5 μl EB buffer (Qiagen), immediately snap-frozen and stored at -80°C until further usage. Gene expression by multiplex qPCR was performed as described (14), but using the primer pairs for 27 target genes and 3 house-keeping genes (POLR2F, SDHA, GNAS) (see **Supplemental Table 4**). Pre-processing and data analysis were conducted using KNIME 4.3.1, R (V4) and RStudio version 1.2.1335. Technical qPCR replicates were averaged. For normalisation to the 3 house-keeping genes the Delta CT method was used (15).

### Statistical Analysis

Differences in proportions of patients or controls cells after scRNAseq and after FACS analysis were evaluated with multiple unpaired T tests in Prism 9. Differences in gene expression analysed by qPCR were calculated by the Wilcoxon test using R (3.5.0). A p-value < 0.05 was considered significant. Cluster Markers and differentially expressed genes were calculated in Seurat using the FindAllMarkers or FindMarkers command, applying a log fold threshold of 0.3 and requiring 30% of the cells to express the marker (13–15).

## Results

### Type I Interferon and Viral Response Genes Are Upregulated in Patients With Mutations in TREX1

**Figure 1:**
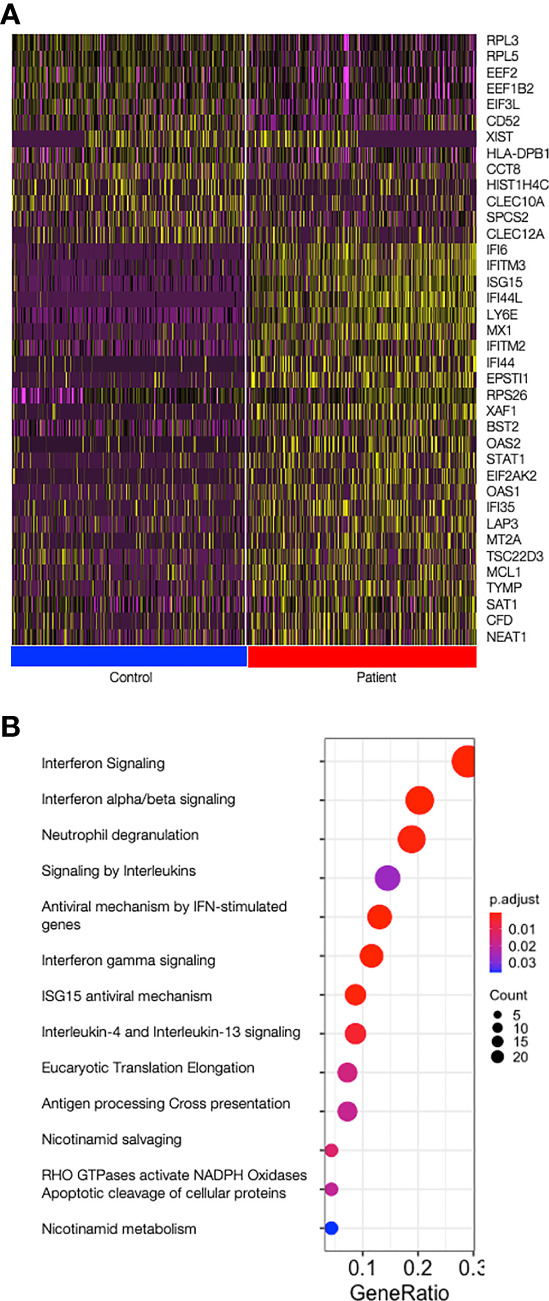
Type I Interferon stimulated genes are upregulated in patients. **(A)** Heat map showing the top 25 most differentially expressed genes between patients and control cells over all cells analysed (all cell types joined); on top, all 13 genes upregulated in controls and on the bottom the top 25 genes upregulated in patients are shown. Control cells are shown on the left (blue bar) and patient’s cells are shown on the right (red bar). Graduation of expression ranges from purple (absent) to yellow (high). **(B)** Dotplot showing significance of the pathways enriched in patients (ReactomePA). Count is the number of genes involved in the pathway. Gene Ratio is the ratio of the genes involved in each pathway to the total number of cluster genes. P adjust is the adjusted p-value.

Monocytes (CD14^+^), cDCs (CD11c^+^CD123^-^), and pDCs (CD11c^-^CD123^+^) were isolated from two patients with mutations in TREX1 and two control individuals and, after pooling the three cell types, subjected to scRNA-seq (**Supplemental Figure 1**). The expression of type I interferon-stimulated genes was increased in patients throughout all cell types (**Figure 1A**, **Supplemental Table 2**). Reactome pathway analysis revealed that upregulated genes in patients were predominantly enriched in pathways related to interferon-, interleukin- and antiviral-signalling (**Figure 1B**).

### A DC Population With Prominent Type I Interferon and Viral Response Gene Profile in Patients With Mutations in TREX1

**Figure 2:**
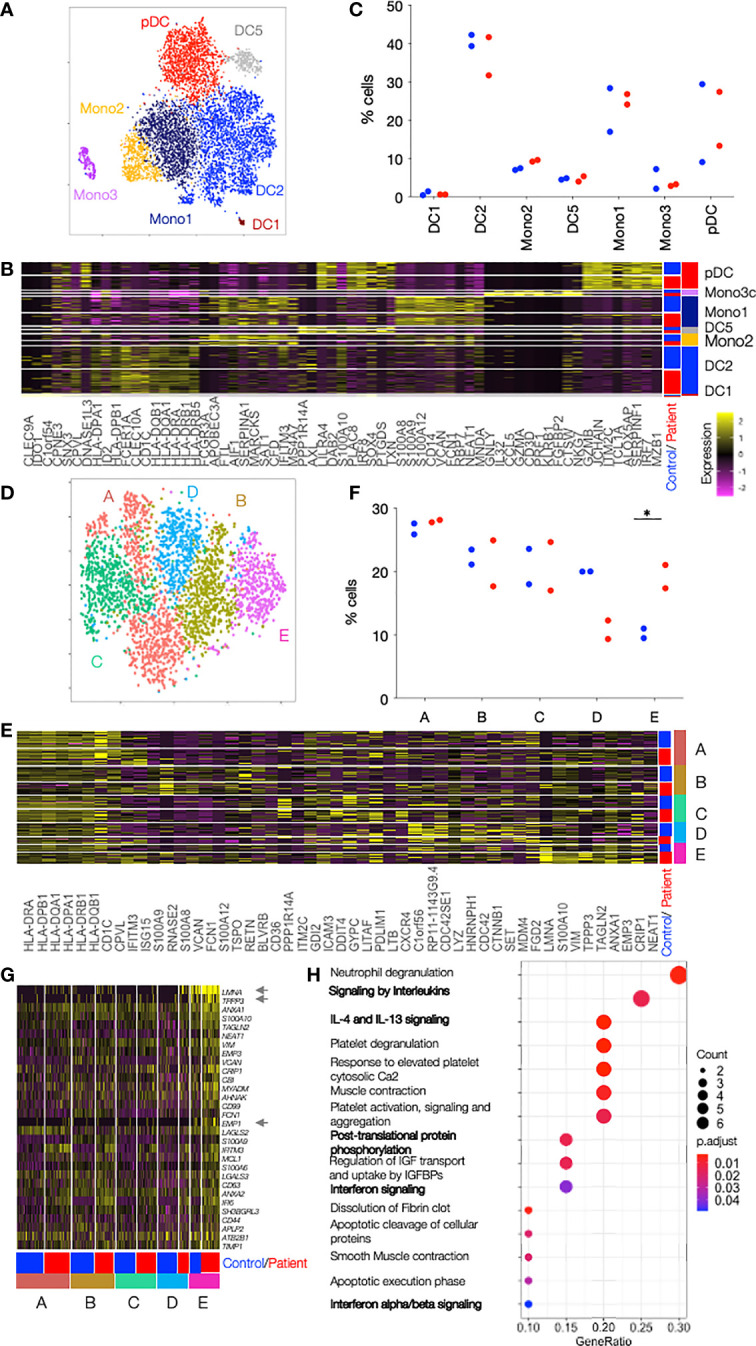
Single cell RNAseq reveals clusters enriched in patient cells. **(A)** DCs and Monocytes isolated from blood of 2 patients with TREX1 mutation and 2 controls were analyzed by single cell RNaseq. tSNE of all sequenced cells after clustering into 7 clusters separates the cells into the 7 cell types annotated and color coded. **(B)** Heat map showing the top 10 cluster markers for each of the cell types. The row color bar on the right shows the color codes (as in A) for the cell types, and the bar on the left shows in blue cells from controls and in red cells from patients. Graduation of expression ranges from purple (absent) to yellow (high). **(C)** Dot plot showing the proportion of patients or control cells found in each cluster. Patients are shown in red and controls in blue. **(D)** tSNE of DC2 cells from 2 patients and controls after clustering into 5 subclusters. **(E)** Heat map showing the top 10 cluster markers for each DC2 subcluster. The row color bar on the right shows the color codes for the subclusters **(A–E)** (as in D), and the bar on the left shows in blue cells from controls and in red cells from patients. **(F)** Dot plot showing the proportion of patients or control cells found in each DC2 subcluster. Patients are shown in red and controls in blue. **(G)** Heat map showing all DC2-E marker genes in cells from all DC2 subclusters (A-E) in controls and patients. All genes belong to the Type I Interferon response according to Interferome. Asterisks point to genes whose expression is almost unique in DC2-E. The row color bar on the right shows the color codes for the subclusters **(A–E)** and the bar on the left shows in blue the cells from controls and in red the cells from patients. **(H)** Dotplot showing the significance of the pathways enriched in cluster DC2-E (ReactomePA). Pathways with relevance for the DCs are in bold. Count is the number of genes involved in the pathway. Gene Ratio is the ratio of the genes involved in each pathway to the total number of cluster genes. P adjust is the adjusted p-value. Comparisons with significance are marked with an *(p-value< 0.05, two-sided t- Comparisons with significance are marked with an *(p-value< 0.05, Mann-Whitney Test).

Unsupervised clustering revealed the presence of previously described cell populations DC1, DC2, Mono2, DC5, Mono1, Mono3 and pDCs, characterised by the expression of, amongst others, CLEC9A and IDO1 (for DC1), CD1c and CLEC10A (for DC2), FCGR3A and APOBEC3A (for Mono2), AXL and LILRA4 (for DC5), CD14 and VCAN (for Mono1), GNLY and CD32 (for Mono3) and GZMB and JCHAIN genes (for pDCs) (12) (**Figures 2A, B**, **Supplemental Table 2**). The frequency of cells in the cell subsets were similar in patients and controls, but with some variation between individuals (**Figure 2C**). The largest DC cluster, DC2, representing around 40% of all cells was further sub-clustered. The two DC2 types previously described as CD1c^+^_A and CD1c^+^_B were found among the 5 identified sub-clusters (12) (**Figures 2D, E**, **Supplemental Table 2**). CD1c^+^_A had high CD1C and HLA gene expression and CD1c^+^_B expressed a number of chronic inflammatory genes such as S100A8 and S100A9. Three other sub-clusters (C, D, E) expressed marker genes that did not allow assignment to any cell types described so far in the literature. Sub-cluster E showed a statistically significant two-fold increase in patient’s (17.3 and 21.0%) as compared to control cells (11.0 and 9.5%) (p=0.04 by unpaired t-test) whereas sub-cluster D, on the contrary, showed enrichment of control cells (20.0 and 19.9%) as compared to patient’s cells (12.3 and 9.3%) (p=0.02 by unpaired t-test) (**Figure 2F**). LMNA, TPPP3 and EMP1 best defined sub-cluster E among all DC2 cells and were almost exclusively expressed in this novel DC2-E population (**Figure 2G**). Of the top 30 genes that identified sub-cluster E, 26 had entries as human Type I Interferon response genes in Interferome (http://www.interferome.org/interferome/home.jspx, v2.0). In addition, “Interferon signaling” and “Interferon alpha and beta signaling” were among the pathways found by the Reactome Pathway analysis (**Figure 2H**). The upregulation of the neutrophil degranulation pathway is most likely driven by the strong upregulation of laminA that has an important role in nuclear evolution in neutrophils (16). Enrichment in genes involved in interferon signaling was also found in sub-cluster A, but not in the other sub-clusters, B, C or D (**Supplemental Figure 2** and **Supplemental Table 3**). Sub-cluster D was characterized by genes important for the HO GTPase signaling (**Supplemental Figure 2C**), a complex pathway regulating activation, pathogen internalization and migration of innate immune cells (17)

### The Novel DC2-E Population Can Be Isolated by FACS Analysis

**Figure 3:**
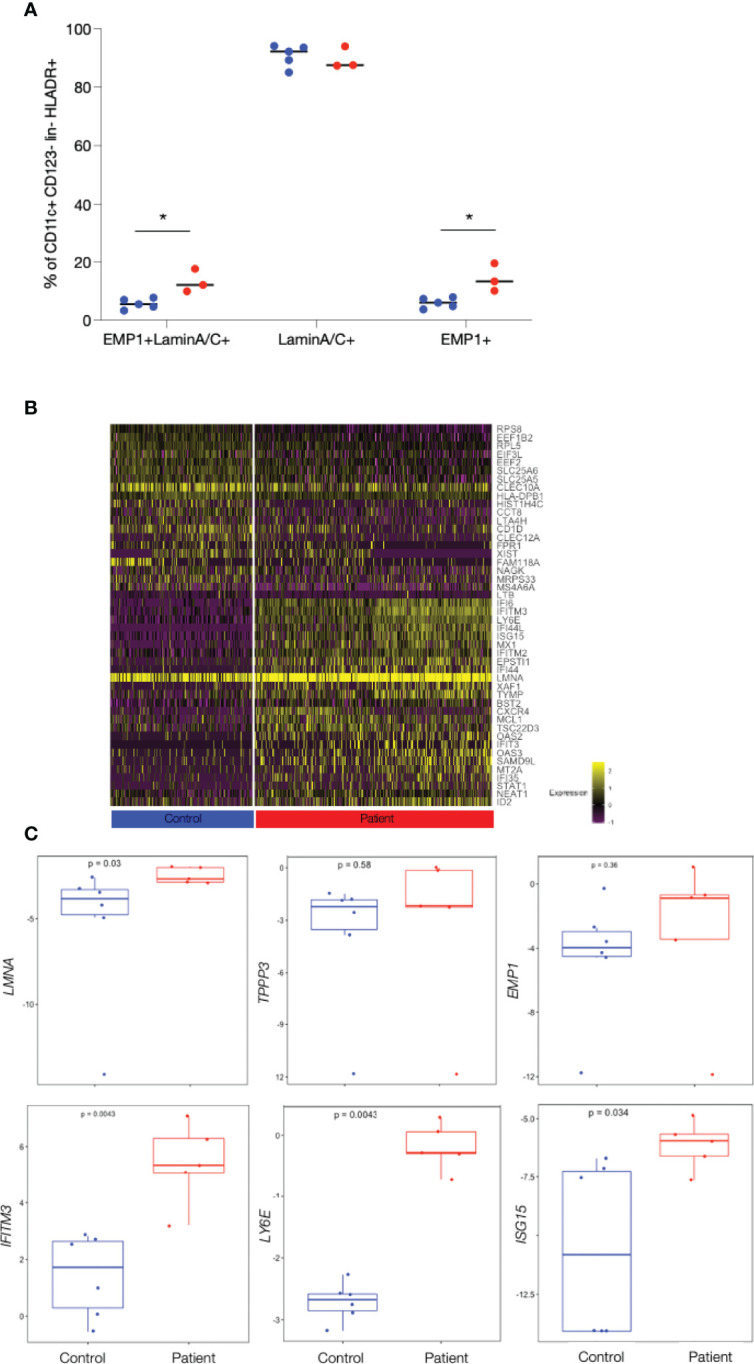
Enrichment of patient’s cells and confirming patient specific gene expression in a CD11c^+^ EMP1^+^ cell subset isolated by FACS. **(A)** Frequency of Lamin A/C ^+^, EMP1^+^ and of Lamin A/C ^+^EMP1^+^ (DC2-E) cells from patients and controls in CD11c+CD123^-^ cells determined by FACS analysis. Controls are shown as blue and patients as red dots. Comparisons with significance are marked with an *(p-value< 0.05, Mann-Whitney Test). **(B)** Heat map showing the top 25 genes differentially expressed between patients and controls in DC2-E cells found by scRNAseq (note that only 21 genes were found upregulated in controls). Genes upregulated and with entries in Interferome are shown in red. The bar below the heat map shows patient’s cell in red (right) and control cells in blue (left). Graduation of expression ranges from purple (absent) to yellow (high). **(C)** Boxplots showing qPCR quantification of exemplary, patient specific genes (found by scRNAseq to be differentially expressed in patients and controls) in sorted CD11c^+^CD123^-^ EMP1^+^ cells batches from patients and controls. The gene analysed is shown on the y-axis and p-values are shown within the plots. Measurements considered to be with significance show a p-value< 0.05, Wilcoxon Rank Sum Test.

To verify the existence of the novel DC2 cell type, DC2-E, PBMCs from the initial 2 patients and 2 controls, from 1 additional patient and from 3 additional controls were stained with a panel containing antibodies against the most exclusive cluster DC2-E markers with available antibodies, LMNA and EMP1 (**Supplemental Figure 4**). A CD11c^+^ CD123^-^ population co-expressing the surface marker EMP1 and the intracellular marker LaminA/C was enriched in patients (mean, 15.2% of CD11c^+^ CD123^-^) as compared to controls (mean, 7.9%; p=0.02) (**Figure 3A** and **Supplemental Figure 4C**). Cells positive for LaminA/C only were present in comparatively higher factions of CD11c^+^ CD123^-^ than the cells double positive for LaminA/C and EMP1 in patients and controls (mean=95.2% and 95.3% of CD11c^+^ CD123^-^, respectively, p=0.97). Cells positive for EMP1 only were as low as the double positive cells but were also higher in patients (mean=15.6% and 8.0% of CD11c^+^ CD123^-^, respectively, p=0.02) (**Figure 3A**). EMP1 and LaminA/C single- and double-positive cells were also found in CD14^+^ Monocytes and pDCs (**Supplemental Figure 3B**). **Figure 3A** indicates that differences between patient and controls were seen when protein expression of EMP1 only was assessed whereas lamin A/C expression was similar between patients and controls (**Figure 3A**). Therefore, we used the surface marker EMP1 to isolate a surrogate DC2-E population by FACS from PBMC of patients and controls (**Supplemental Figure 4**) and used qPCR to show that the isolated CD11c^+^ CD123^-^EMP1^+^ population was enriched for the mRNA of LMNA and EMP1 (**Supplemental Figure 5**) suggesting that a population at least closely resembling DC2-E can be isolated by FACS. The specificity of the population was further validated by qPCR analysis of additional cluster markers (**Figure 2G**). 19 out of 20 tested markers were expressed at a higher level in the DC2-E population than in DC2 cells or monocytes (**Supplemental Figure 5** and **Supplemental Table 4**).

To further analyse the DC2-E population we analysed the genes upregulated in patients and controls in this subpopulation in more detail (**Figure 3B**). 64 genes were found to be upregulated in patients as compared to controls by scRNAseq (**Figure 3B**, **Supplemental Table 2**) in DC2-E and 32 thereof had entries as human type I interferon response genes in Interferome (**Figure 3B**). Importantly, we quantified 17 of these genes by qPCR and confirmed 9 to be significantly elevated in patients in the FACS sorted surrogate DC2-E population (**Figure 3C** and **Supplemental Table 4**).

Further analysis revealed significantly more CD11c^+^CD123^-^EMP1^+^ laminA/C^+^ (DC2-E) cells expressing the activation markers CD80 and CD86 in patients as compared to controls (**Supplemental Figure 6**) (CD80, mean=4.6% versus 2.1%, p= 0.047 and CD86, 92.9% versus and 87.4% p=0.045), but the median expression intensity of the activation markers CD40, CD80 and CD86 was similar in these cells in patients and controls.

## Discussion

Here, we describe a novel myeloid DC population enriched in lupus patients with TREX1 mutations. This DC population is characterized by strong expression of in interferon stimulated genes potential diagnostic. The general role of DCs as antigen presenting cells is well established but a specific function of myeloid DC and subpopulations in autoimmunity is less described. We were able to identify a specific DC subpopulation in patients with familial chilblain lupus using scRNAseq. This population was found by sub- clustering the CD11c^+^ CD1c^+^ myeloid DC population designated as DC2 (or CD1C^+^_A + CD1C^+^_B) by Villani et al (12) and therefore represents a subpopulation of cDC2 in the classical nomenclature (10) (see **Supplemental Figure 1**). We confirmed the presence of this new population in a second cohort by flow cytometry and we were able to sort this subpopulation using defined surface markers.

The identified DC2-E subpopulation was enriched among myeloid DC2 cells of TREX1 deficient patients and defined by the expression of LMNA (lamin A/C) and EMP1. Lamin A/C is an essential component of the nuclear envelope and upregulation of LMNA is a feature of mature DCs (18). LMNA might be induced by type I IFNs as protective cellular antiviral response to prevent egress of viral particles or chromatin from the nucleus (19). Lamin A/C- chromatin interaction has been described to prevent HIV-1 transcription and sustain viral latency (20). Our data suggest LMNA mRNA expression to be a specific antiviral response to chronic type I IFN activation in DC2-E. LMNA upregulation might also be a response mechanism to stress and DNA damage in TREX1 deficient cells aiming in decreasing the extent of nuclear membrane rupture (6, 21).

The function of EMP1 in DCs has not yet been defined. It has been described in cancer cells involved in migration and adhesion (22). Here, it was especially important because staining for EMP1 protein expression on the cell surface facilitated isolation of the DC2-E population from PBMC. The functional role of the DC2-E subpopulation needs further exploration. However, cells expressing the costimulatory molecules CD80 and CD86 were slightly enriched in the DC2-E population in patients versus controls, suggesting a possible role for these cells in T cell priming and activation. This feature is shared with the population of monocytes and CD11c+ DCs from patients with TREX1 mutation and might indicate the activated phenotype of antigen presenting cells in patients.

Disease specificity in gene expression was detected in myeloid DCs but not in pDCs. This might correlate with the proposed functional role for myeloid but not plasmacytoid DCs in TREX1 deficient mice (8). The downregulation of TREX1 in CD11c^+^ myeloid DCs was sufficient to induce autoimmunity in mice highlighting the importance of myeloid DCs for the induction of autoimmunity (8).

The limitations of this study include the small number of patients analysed. Follow up studies enrolling larger cohorts and cohorts with other interferonopathies will enable to overcome this.

Further analyzing type I IFN-responding cell subtypes in monogenic types of lupus will help understanding the development of autoimmunity due to TREX1 mutations and will shed light on other types of SLE induced by similar disturbance in intracellular nucleic acid metabolism. These mechanisms are potentially important also in complex cases of SLE and their pathogenic exploration will help to define more targeted therapies.

## Data Availability Statement

The datasets generated for this study has been deposited at the European Genome-Phenome Archive (EGA) and can be accessed through accession number EGAS00001006215.

## Ethics Statement

The studies involving human participants were reviewed and approved by Ethikkommission an der Technischen Universität Dresden. The patients/participants provided their written informed consent to participate in this study.

## Author Contributions

AE was involved designing the studies, conducting experiments, acquiring data, analyzing data and writing the manuscript; DM, SR, AL and NZ conducted experiments; AG acquired and analyzed data; MA designed the studies; EB, SB and CG were involved in study design and manuscript writing. All authors contributed to the article and approved the submitted version.

## Funding

This work was supported by the Deutsche Forschungsgemeinschaft (German Research Foundation), grant TRR237 369799452/404458960, KFO 249/GU1212/1-1 and 1-2 to CG and a CRTD grant to CG and AE and funding to DFG Research Center and Cluster of Excellence–Center for Regenerative Therapies Dresden (FZ 111 to AE, DM, MA and EB).

## Conflict of Interest

The authors declare that the research was conducted in the absence of any commercial or financial relationships that could be construed as a potential conflict of interest.

## Publisher’s Note

All claims expressed in this article are solely those of the authors and do not necessarily represent those of their affiliated organizations, or those of the publisher, the editors and the reviewers. Any product that may be evaluated in this article, or claim that may be made by its manufacturer, is not guaranteed or endorsed by the publisher.
