# Corrigendum: A novel type I interferon primed dendritic cell subpopulation in TREX1 mutant chilblain lupus patients

**DOI:** 10.3389/fimmu.2022.1094578

**Published:** 2022-11-25

**Authors:** Anne Eugster, Denise Müller, Anne Gompf, Susanne Reinhardt, Annett Lindner, Michelle Ashton, Nick Zimmermann, Stefan Beissert, Ezio Bonifacio, Claudia Günther

**Affiliations:** ^1^ Center for Regenerative Therapies Dresden, Faculty of Medicine, Technische Universität Dresden, Dresden, Germany; ^2^ Center for Molecular and Cellular Bioengineering (CMCB), DRESDEN-Concept Genome Center, Technische Universität Dresden, Dresden, Germany; ^3^ Department of Dermatology, Faculty of Medicine, University Hospital Carl Gustav Carus, Technische Universität Dresden, Dresden, Germany; ^4^ Faculty of Medicine, Paul Langerhans Institute Dresden of Helmholtz Centre Munich at University Clinic Carl Gustav Carus, Technische Universität Dresden, Dresden, Germany

**Keywords:** monogenic familial chilblain lupus, SLE, Type I interferons, dendritic Cells (DC), LMNA, Lamin A/C, Trex1

In the published article, there was an error in affiliations 3 and 4. Instead of “[^3^ Faculty of Medicine, Paul Langerhans Institute Dresden of Helmholtz Centre Munich at University Clinic Carl Gustav Carus of Technische Universität (TU) Dresden, Dresden, Germany, ^4^ Department of Dermatology, Faculty of Medicine, University Hospital Carl Gustav Carus of Technische Universität (TU) Dresden, Dresden, Germany]”, it should be as shown above.

The authors apologize for this error and state that this does not change the scientific conclusions of the article in any way. The original article has been updated.

## Conflict of interest

The authors declare that the research was conducted in the absence of any commercial or financial relationships that could be construed as a potential conflict of interest.

## Publisher’s note

All claims expressed in this article are solely those of the authors and do not necessarily represent those of their affiliated organizations, or those of the publisher, the editors and the reviewers. Any product that may be evaluated in this article, or claim that may be made by its manufacturer, is not guaranteed or endorsed by the publisher.

