# Supplementary figures and images for: A Novel Type I Interferon Primed Dendritic Cell Subpopulation in TREX1 Mutant Chilblain Lupus Patients
Source: Front Immunol. 2022 Jul 13;13:897500. doi: 10.3389/fimmu.2022.897500 (PMC9327789; doi:10.3389/fimmu.2022.897500)

Supp Figure 1

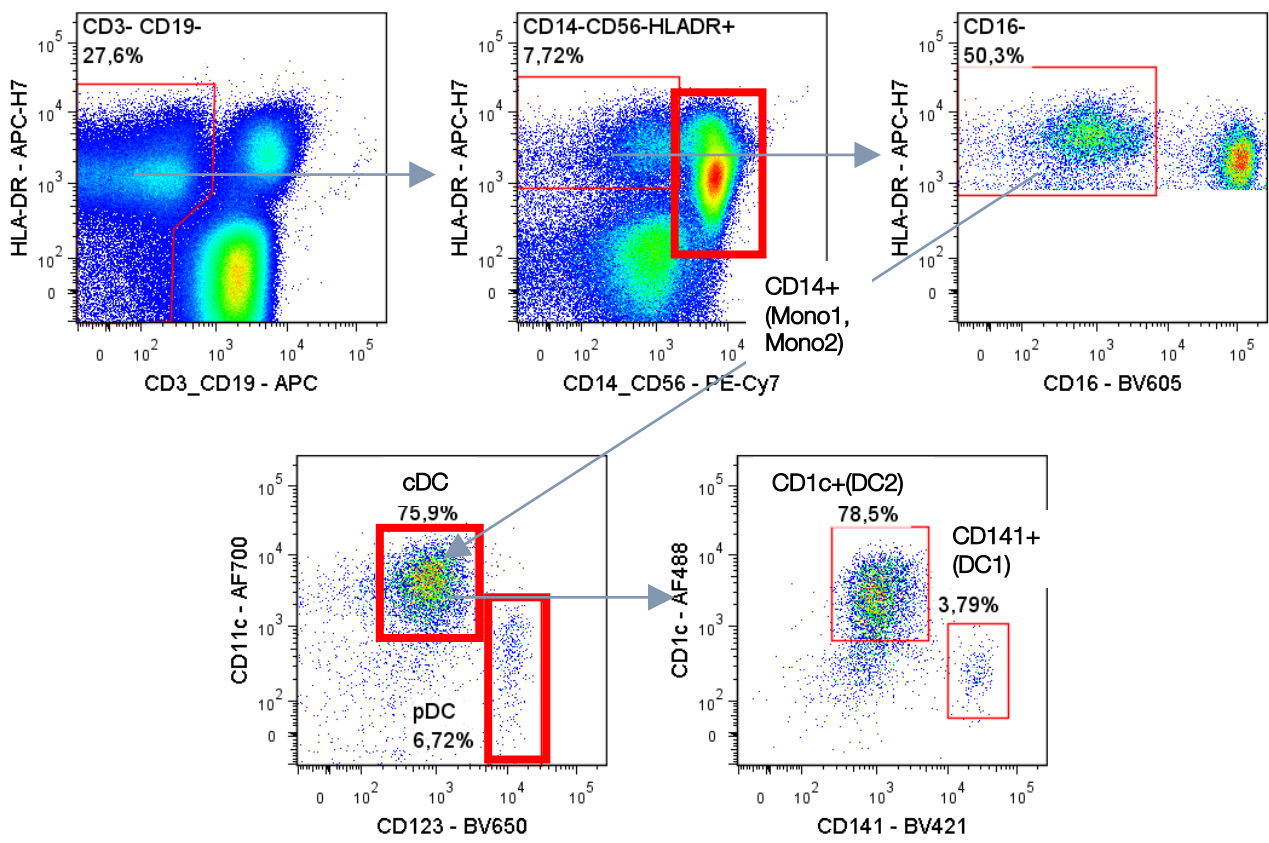

Supp Figure 2

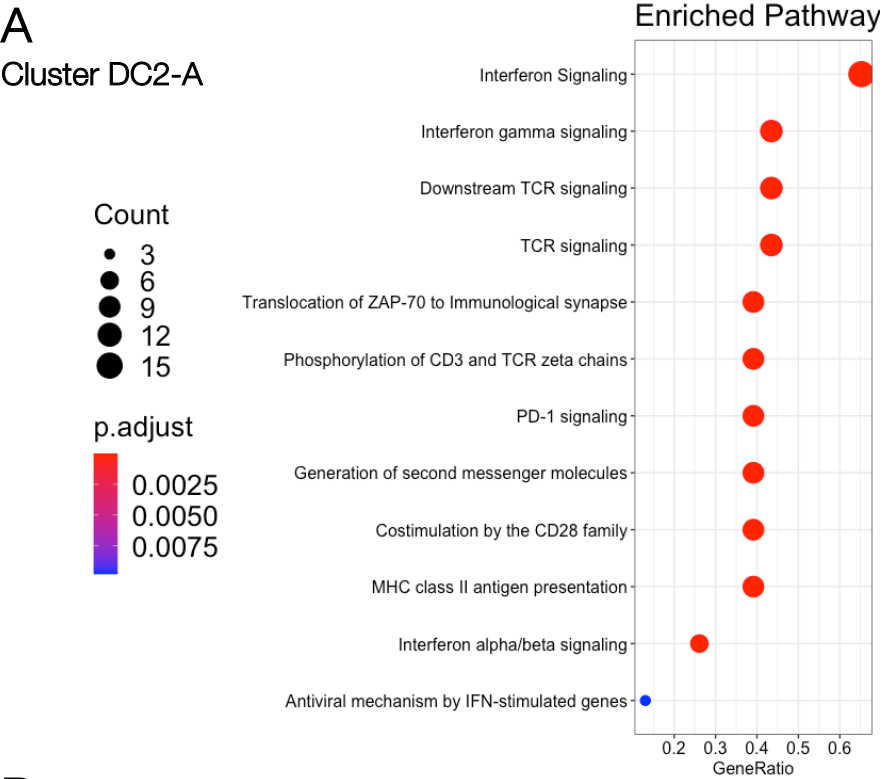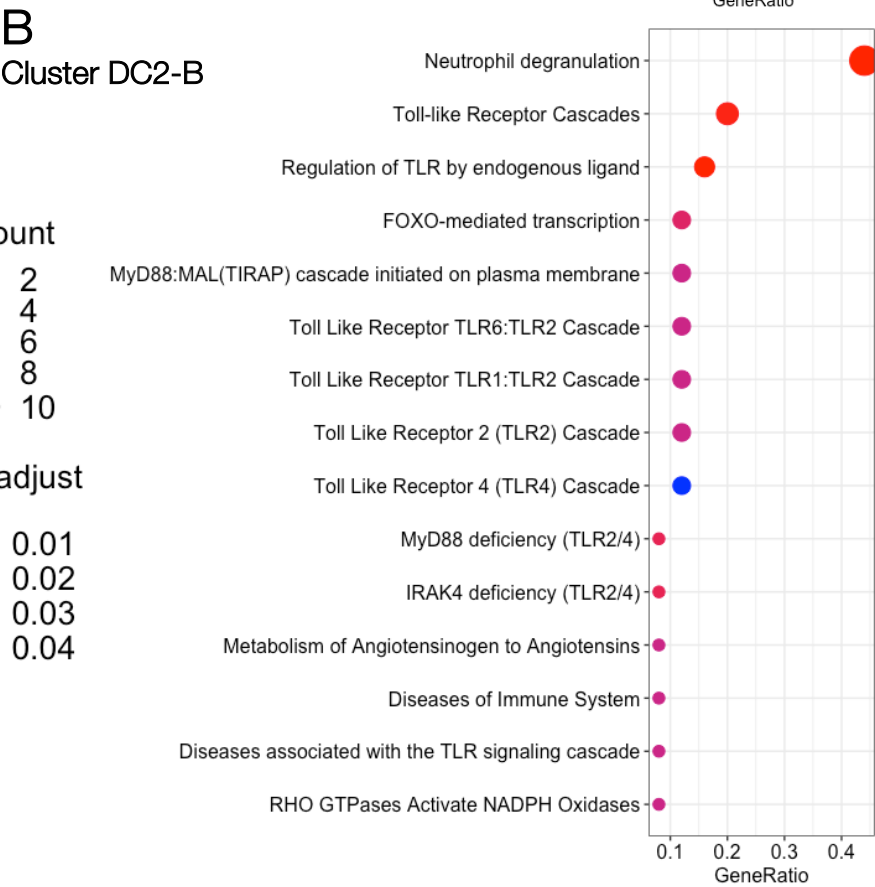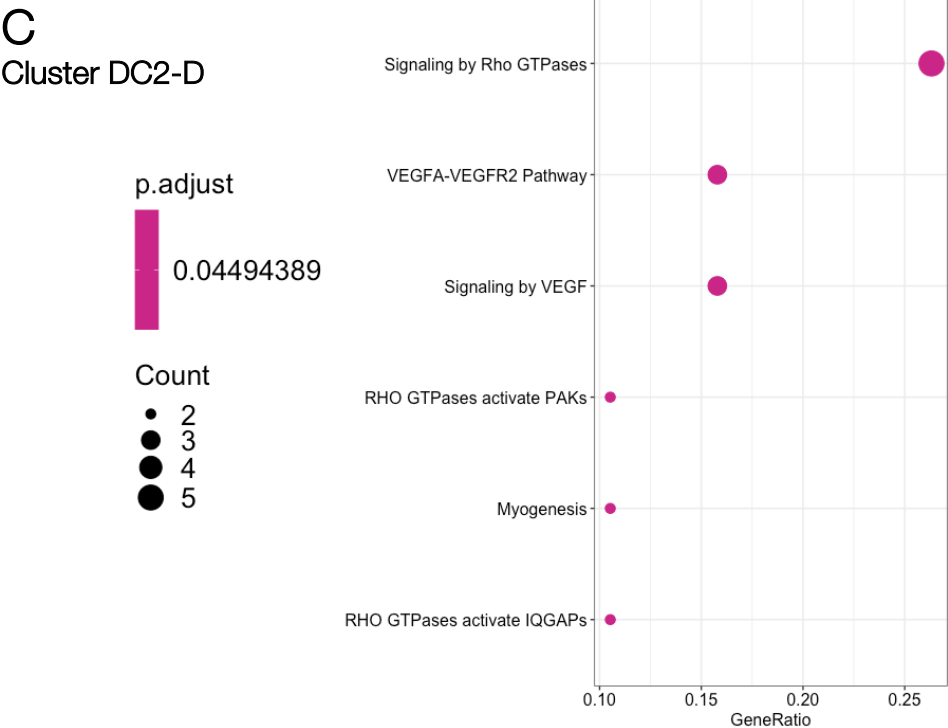

Supp Figure 3

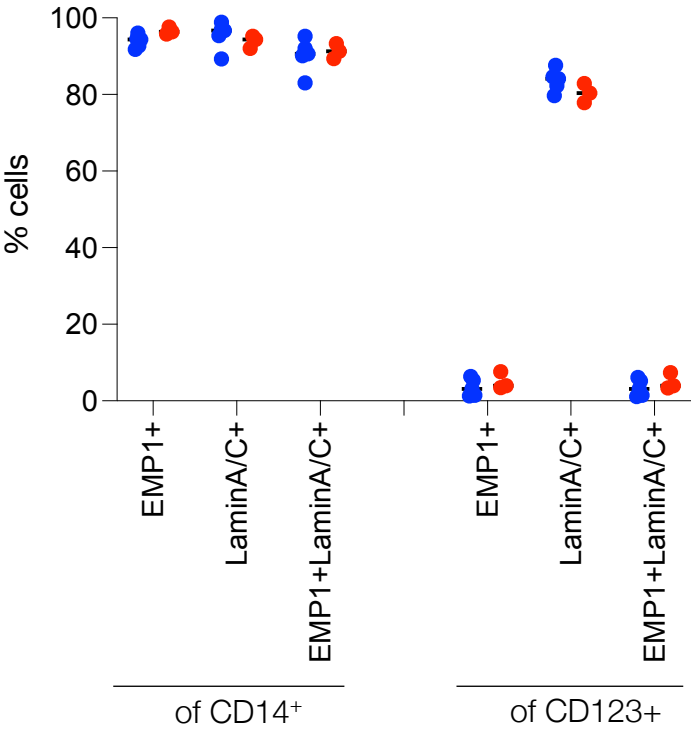

Supp Figure 4

A

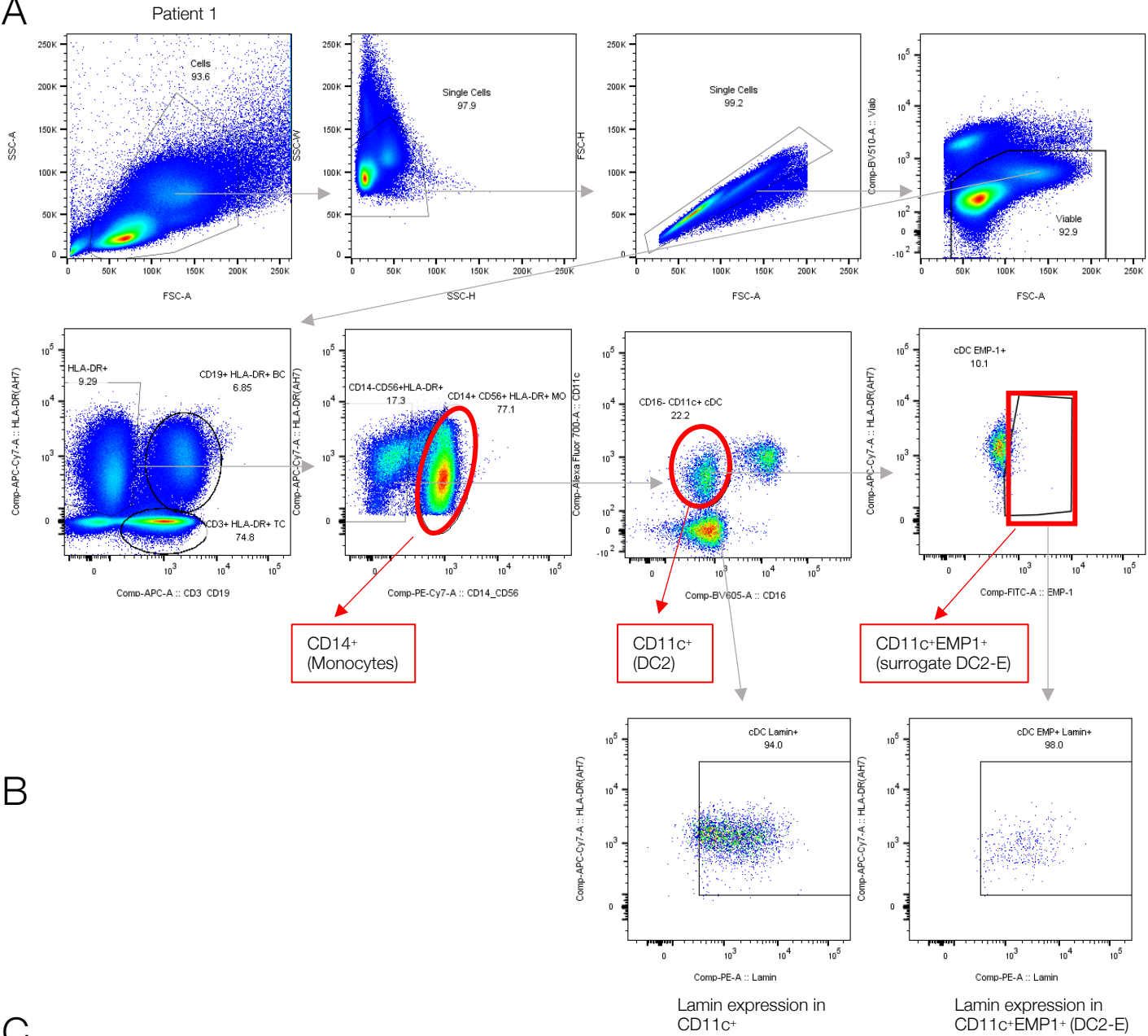

B

C

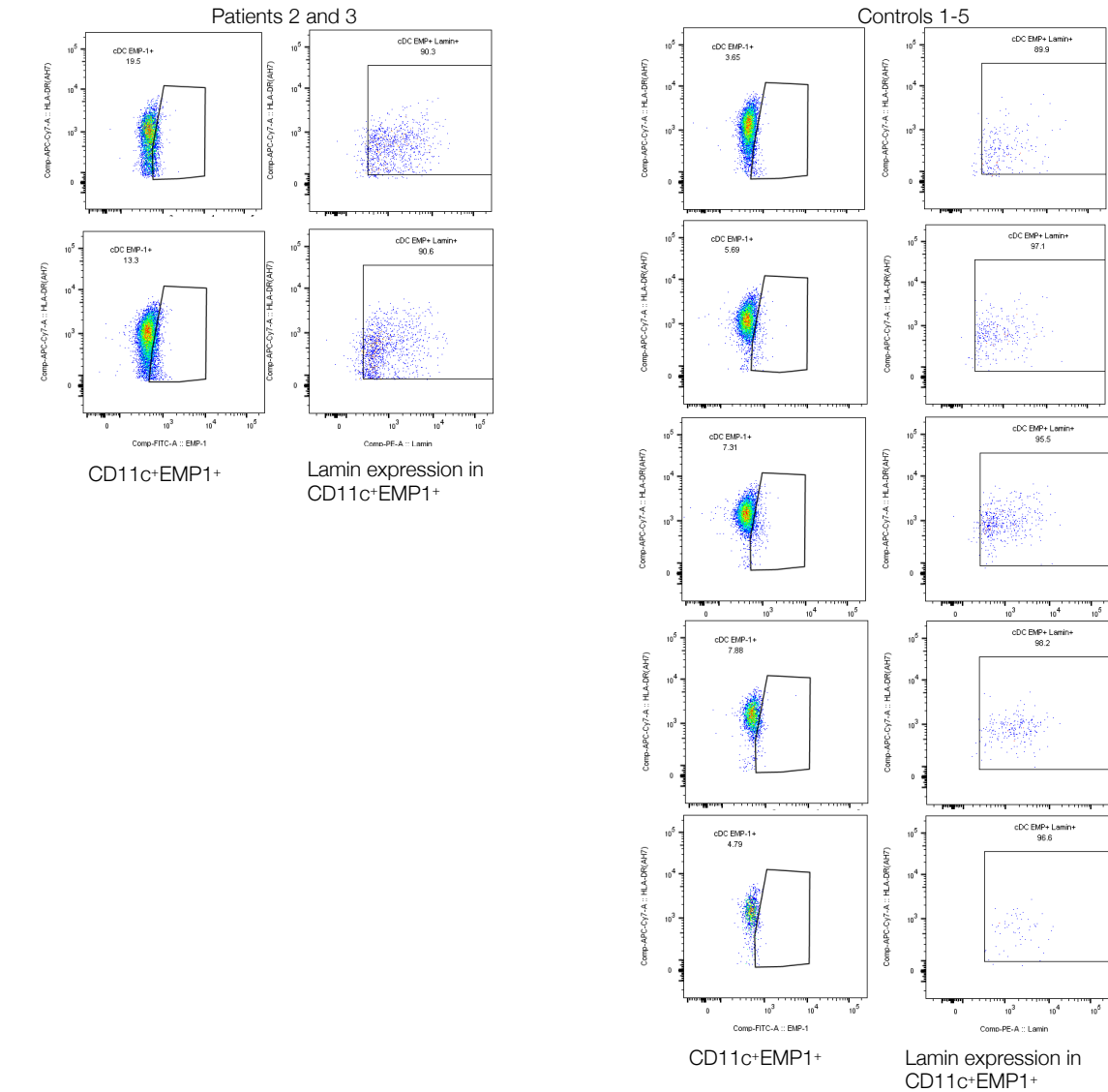

Supp Figure 5

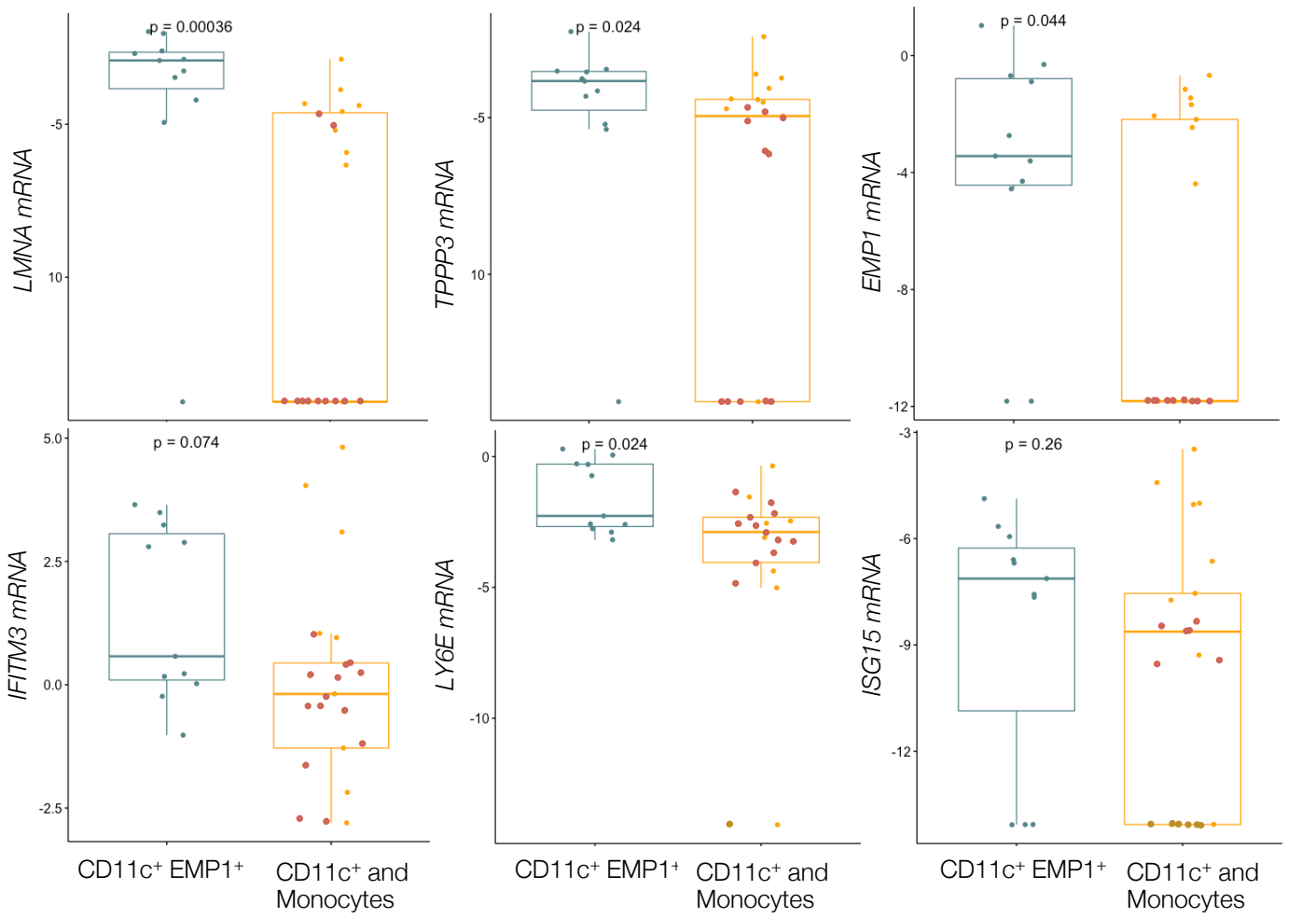

Supp Figure 6

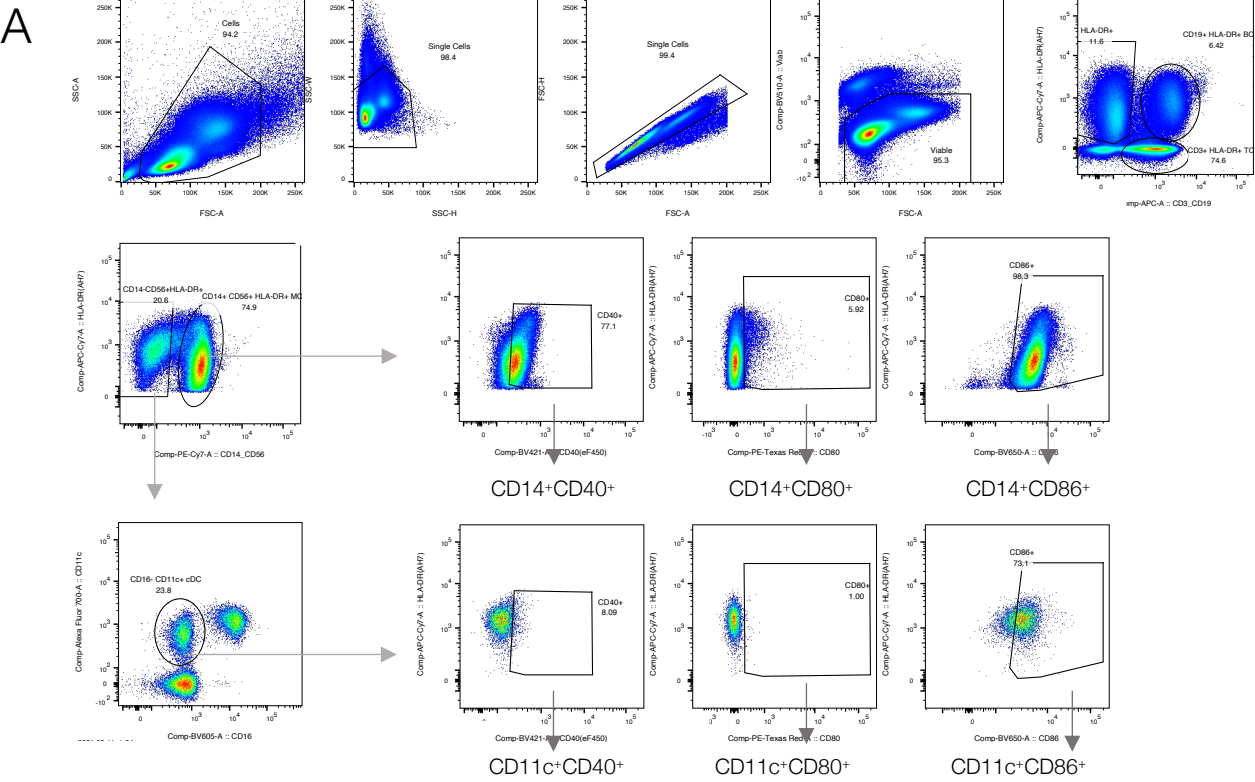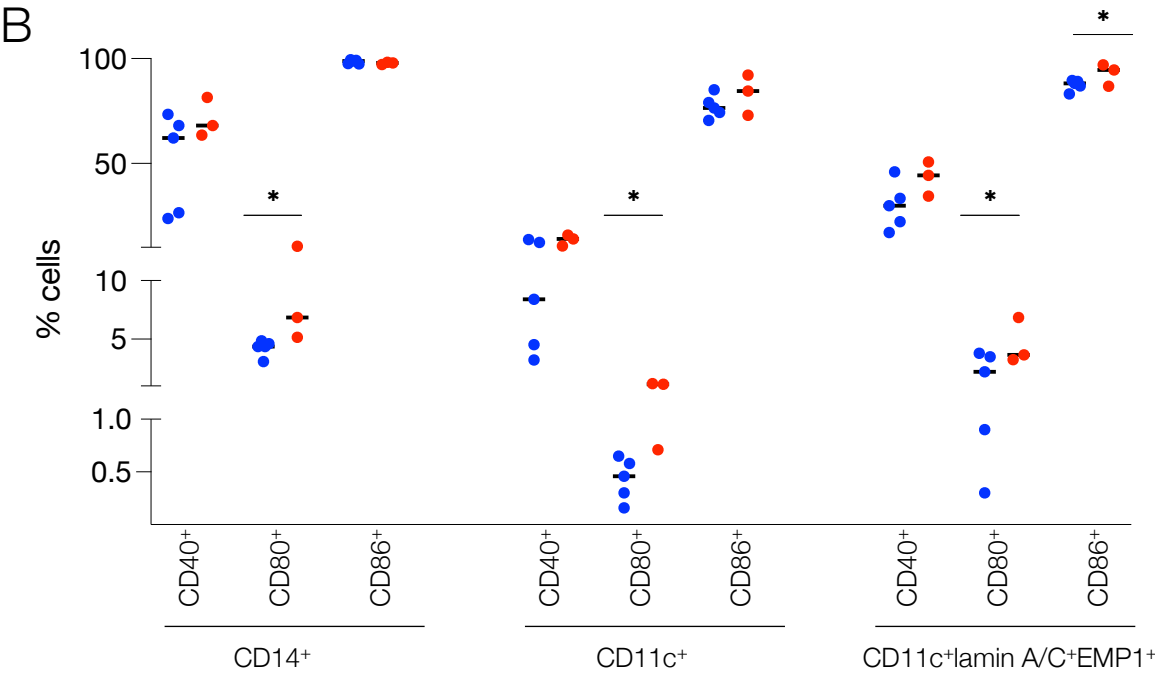

Supplement: Supplementary file 1 [file DataSheet_1.pdf]
